# Early career challenges of physical education teachers

**DOI:** 10.3389/fpsyg.2026.1957218

**Published:** 2026-10-30

**Authors:** Sevilay Dilmen, Hidayet Suha Yuksel

**Affiliations:** 1 Graduate School of Educational Sciences, Ankara University, Ankara, Türkiye; 2 Department of Physical Education and Sports, Faculty of Sport Sciences, Ankara University, Ankara, Türkiye

**Keywords:** early career challenges, physical education teacher education (PETE), physical education teachers, professional adjustment, psychosocial dynamics, reality shock

## Abstract

**Introduction:**

Early career physical education teachers face challenges similar to those of other novice teachers, in addition to field-specific difficulties. Their professional adjustment depends on the interaction between personal psychological and pedagogical resources and the workplace demands. Successfully navigating this process and ensuring adequate socio-institutional support are essential for career development and sustained professional practice. The aim of this study is to reveal the career challenges faced by physical education teachers in their first 5 years of the profession and to examine how these challenges are reflected in their professional adaptation processes.

**Methods:**

This study, conducted using a qualitative research approach, employed a holistic single-case study design. Semi-structured interviews were conducted with 25 teachers (11 women, 14 men) selected through criterion sampling, and the data were analyzed using inductive content analysis.

**Results:**

The findings are structured under three main themes: (1) Regional and Psychosocial Dynamics, (2) Physical Education Teacher Education (PETE) and Perceived Professional Competence, and (3) Stakeholder Relationships and Perceived Social Support. First, teachers’ accounts reveal pronounced adaptation difficulties tied to the climatic, transportation, and local cultural characteristics of their working environments. Second, participants highlighted significant operational hurdles in administrative tasks, classroom management, and student assessment, reporting that the theory-practice gap in undergraduate education created a perceived lack of readiness for pedagogical and sociocultural complexities. Third, a perceived lack of administrative support, some students’ view of the course as mere leisure, and some parents’ performance-driven attitudes were described as factors eroding the necessary social support systems.

**Discussion:**

As a result, physical education teachers in the early stages of their careers undergo a professional adjustment process that involves complex and multilayered challenges at the intersection of structural, pedagogical, geographic, and sociocultural dynamics. By integrating these challenges into a single, holistic case study, this research expands upon previous studies on career entry and physical education—which typically examine these factors independently—and offers a comprehensive perspective on the interrelated challenges and adaptation processes experienced by physical education teachers in the context of Türkiye.

## 1 Introduction

The early career phase of teachers is demanding, during which they construct their professional identities and invest considerable effort in adapting to the profession. In the context of teaching, the “early career period” typically refers to the first few years following entry into the profession and is generally defined in the literature as spanning approximately the first 1–5 years. In Huberman’s (1989) teacher career cycle model, the first 5 years, encompassing the “survival” and “stabilization” stages, are regarded as a critical phase in which teachers develop and consolidate their professional identities (Beijaard et al., 2004). Analyses based on TALIS 2018 data distinguish two specific subgroups within novice teachers: “beginning teachers” (0–2 years of experience) and “advanced beginners” (2–5 years of experience). This categorization likewise indicates that the early career period is, in broad terms, completed within 5 years (Admiraal et al., 2023). Throughout this phase, novice teachers face a wide range of psychological, pedagogical, and social challenges. High workload, disruptive student behavior, role conflict, adjustment problems, adaptation to school culture, alignment with instructional approaches, and meeting professional expectations are among the principal difficulties observed in the initial years of teaching (Gu and Day, 2007; Gu and Li, 2013; Xue, 2021). In addition, more specific challenges, such as establishing effective communication with students, collaborating with parents, and working with students with special educational needs, represent further significant sources of difficulty for beginning teachers (Gu, 2024; Škoda et al., 2025). However, the challenges faced by teachers entering the profession are not limited to pedagogical issues; they also require substantial psychological and emotional resilience. For almost all teachers, the first years of their careers are regarded as the most difficult period of their lives (Barkauskaitė and Meškauskienė, 2018). Factors such as stress, problems in maintaining work–life balance, feelings of isolation, uncertainty, and insufficient support place considerable strain on the resilience of teachers during their early career years (Jin et al., 2025). Because such difficulties increase the risk of attrition at the outset of teachers’ careers, the initial years can be considered critical for their adjustment and professional development (Whitford, 2020). Overall, the challenges encountered in the early years of teaching appear to be multidimensional and complex, and the adaptation process requires support that extends beyond individual effort. In particular, it should be reinforced through strategies such as mentoring provided within the school environment, access to professional development opportunities, social support, and careful attention to teachers’ pedagogical needs (Gareis and Nussbaum-Beach, 2007; Jin et al., 2025; Martin et al., 2016; Toom and Husu, 2021; Whitford, 2020).

The challenges encountered by physical education teachers in the early years of their careers, while comparable to the difficulties experienced during the induction period of the teaching profession in general, also encompass specific challenges arising from the distinctive nature of the field. Owing to their structure and nature, physical education lessons require a different analytical perspective from other school subjects. In particular, the fact that the instructional setting differs from standard classrooms, the type of equipment employed, and factors such as clothing distinguish physical education lessons from other courses and may likewise differentiate the challenges experienced by teachers. Overall, like the difficulties faced by other teachers, physical education teachers encounter problems stemming from stakeholders (administration, colleagues, parents) and related to instructional practices and students (Demirhan et al., 2014; Sáenz-López et al., 2011; Ulucan et al., 2012). Beyond these general challenges, one specific area in which physical education teachers have trouble is the process of adjustment in classroom management and communication with students (Poteliūnienė and Šatikauskaitė, 2015). Although classroom management is a domain in which many teachers struggle during the early stages of their careers, it can become even more demanding in the context of physical education because of the characteristics of the learning environment. Furthermore, the psychological and physical changes associated with adolescence may manifest as disengagement from or negative attitudes toward physical education lessons for some students, thereby complicating the professional adaptation of novice physical education teachers. In the study conducted by Öztürk Karataş (2021), students’ failure to bring sports equipment and their resistance to participating in lessons were identified as the most significant challenges encountered. Similarly, Temiz and Sivrikaya (2021) revealed that novice physical education and sports teachers did not feel adequately competent in areas such as professional competence, classroom management, subject-matter knowledge, guidance, teaching methods and techniques, and the use of instructional materials and equipment, and that their undergraduate education did not meet their expectations in terms of practical professional preparation.

Considering that the challenges encountered in the early years of the profession are decisive for the career development of physical education teachers, properly managing this adaptation process and establishing the necessary support mechanisms is vital. When they enter the profession, physical education teachers may be assigned to work under different conditions. The fact that physical education teachers are often appointed to rural areas at the beginning of their careers can be regarded as a factor that influences both the sociocultural environment they encounter and the school’s resources in terms of equipment and facilities. In this sense, the real-life experiences of teachers, who mostly complete teaching practice (internship) in urban schools during their undergraduate studies, may be insufficient for adapting to this diversity. Therefore, physical education teacher education (PETE) programs need to be diversified in such a way that, beyond teaching different skills to prospective teachers, they provide multidimensional experiential opportunities that encompass the dynamics of different types of schools and communities (Flory, 2016). An examination of teachers’ narratives about their professional experiences shows that, despite having undergone an intensive pre-service training process, the first day of teaching remains a particularly powerful and distinctive memory in their minds. Although pre-service teachers develop a theoretical foundation and gain practical experience during their undergraduate education, the first moment when they are left alone in the classroom is often associated with a strong sense of anxiety and uncertainty (Kozikoğlu and Senemoğlu, 2018). In this context, while the development of undergraduate education plays a critical role in supporting adaptation processes during the early career phase, the inherently variable nature of professional practice and the realities of the field turn this adaptation process into a unique experience that is, to some extent, independent of undergraduate training. In this regard, mentoring from experienced teachers during entry into the profession emerges as an important resource for helping physical education teachers overcome the challenges they face. Despite this importance, factors such as administrative pressure, unfavorable working conditions, and insufficient collegial support may act as barriers to this process (Poteliūnienė and Šatikauskaitė, 2015). Given that senior teachers are thought to possess a greater capacity to cope with professional challenges, concrete support mechanisms are needed for teachers at the beginning of their careers. In this regard, the establishment of school-based mentoring programs, enhancement of administrative support, and updating of undergraduate curricula stand out as key avenues for improving and supporting professional adjustment (Poteliūnienė and Šatikauskaitė, 2015; Flory, 2016).

The fact that more experienced teachers have lower stress levels and face burnout problems less frequently underscores the importance of focusing on the difficulties that physical education teachers encounter in their professional adjustment during the early stages of their careers (Ulucan et al., 2012). The professional adaptation processes of teachers in the early career period may be influenced by the balance between the psychological and pedagogical resources they possess and the demands of the environment in which they work. Given that this is the period in which teachers construct their professional identities, and that it is decisive for their levels of burnout and their decisions about whether to remain in the profession, accurately analyzing the dynamics of this phase is of critical importance (Beijaard et al., 2004; Feiman-Nemser, 2001). In this context, the main aim of this study is to reveal the nature and implications of the challenges experienced by physical education teachers in the early years of their careers, with particular attention to how these challenges influence their professional adaptation. In line with this aim, the central research question has been formulated as follows: ‘What challenges—particularly those related to regional and sociocultural conditions, PETE experiences, and relationships with school stakeholders—do physical education teachers in the early stages of their careers face during the process of entering the profession, and how are these challenges reflected in their professional adaptation processes?’

## 2 Materials and methods

Qualitative research conceptualizes social reality not as a fixed, researcher-independent data field but as a phenomenon constructed within context and rendered meaningful through interpretation. Accordingly, the researcher is positioned not merely as a data collector but as a co-constructor of meaning with participants who interpret this meaning reflexively (Drisko, 2025; Watson, 2024). In the present study, this epistemological stance guided both the design of the semi-structured interviews and the inductive content analysis, with the aim of co-constructing an in-depth understanding of early career challenges in their specific sociocultural context. In this sense, qualitative findings are not generalizable; rather, through detailed contextual descriptions, theoretical transparency, and intensive interpretation, they offer conceptual insights that can inform the understanding of other situations (Drisko, 2025; Smith, 2018). Therefore, the findings of the present study should be interpreted in light of the specific characteristics of the 25 teachers interviewed, the predominance of schools in Eastern and Southeastern Anatolia, and the use of online interviews, which together may limit the transferability of the results to other regions or educational systems.

This study employed a holistic single-case design. The “case” was defined as “the early-career challenges faced by physical education teachers working in public schools across various regional contexts in Turkey during their first five years of service.” Although the participants work in different provinces, they share a clearly defined temporal boundary (1–5 years of professional seniority) and common contextual characteristics (working in public schools, mostly in rural areas). This holistic design conceptualizes physical education teachers’ professional adaptation process as a comprehensive structure comprising professional experiences, structural constraints, and encountered challenges. The case study methodology allows for an in-depth examination of a well-defined phenomenon within its natural context; in this way, it reveals both elements specific to that case and patterns of meaning that can be transferred to similar contexts (Baxter and Jack, 2008; Merriam, 2010). As Creswell and Poth (2016) also note, this approach possesses a critical capacity for exploring under-researched phenomena in their natural settings and providing a detailed thematic analysis. In this context, this design was chosen to thoroughly examine how regional, organizational, and interpersonal dynamics shape the challenges faced during the early stages of a career.

### 2.1 Participants

The study participants consisted of 25 physical education teachers working in different regions of Türkiye, with up to 5 years of professional experience, who voluntarily participated in the study. In selecting the participants, a criterion-based sampling method was used, which relies on identifying individuals who meet predefined criteria. In this context, the primary criteria were that participants be permanently employed at a public school and be within the first 5 years of their professional careers. A total of 25 physical education teachers (11 women and 14 men) participated in the study. The provinces where the participants were employed were distributed as follows: Adıyaman (2), Ağrı (2), Ankara (4), Ardahan (2), Gaziantep (2), Hakkari (1), Istanbul (1), Muş (4), Şanlıurfa (4), Van (2), and Zonguldak (1). As seen in the distribution, the majority of participants were assigned to the Eastern and Southeastern Anatolia regions—a concentration shaped by structural dynamics such as high teacher turnover, initial assignment quotas, and mandatory service obligations. Focusing on this context enables the study to shed light on the challenges faced at the beginning of their careers—challenges that are particularly pronounced but may not be sufficiently documented—in schools that are in need and exhibit rural and sociocultural diversity. Detailed information regarding the participants’ demographic characteristics and professional experience is presented in Table 1.

**Table 1:** Demographic and professional profile of the participants.

Participant	Age	Gender	Teaching experience	Number of assigned schools	School level	City
1	24	Female	1 year	1	Middle school	Şanlıurfa
2	24	Male	1 year	1	Middle school	Van
3	25	Male	1 year	1	Middle school	Adıyaman
4	25	Male	1 year	1	Middle school	Hakkari
5	25	Female	2 years	1	Middle school	Şanlıurfa
6	26	Male	3 years	3	Middle school	Adıyaman
7	26	Male	1 year	1	Middle school	Ardahan
8	26	Female	3 years	2	High school	İstanbul
9	26	Male	2 years	1	Middle school	Muş
10	26	Female	1 year	2	Middle school	Şanlıurfa
11	26	Male	3 years	1	Middle school	Van
12	27	Female	3 years	2	Middle school	Gaziantep
13	27	Female	1 year	1	Middle school	Gaziantep
14	27	Male	3 years	3	Middle school	Muş
15	28	Female	5 years	1	Middle school	Ankara
16	28	Female	4 years	1	Middle school	Şanlıurfa
17	29	Female	1 year	1	Middle school	Ankara
18	29	Male	4 years	1	Middle school	Zonguldak
19	30	Male	4 years	1	High school	Ağrı
20	30	Male	5 years	1	Middle school	Muş
21	30	Male	4 years	1	Middle school	Muş
22	32	Male	1 year	2	Middle school	Ağrı
23	33	Male	5 years	2	Middle school	Ardahan
24	34	Female	5 years	2	Middle school - High school	Ankara
25	34	Female	4 years	2	Middle school - High school	Ankara

In accordance with the Ministry of National Education’s (MONE) research and field access procedures, a two-stage approach was followed in forming the sample group. In the first stage, taking into account the MONE’s authorization system, schools were identified by reaching out to teachers who met the specified criteria and were part of the researchers’ professional network; in the second stage, the names of these schools were submitted to the MONE to obtain the necessary legal authorizations. Since the sample group was predetermined through this meticulous process prior to the interviews and direct communication was established with the participants, no one withdrew from the study or abandoned the interview midway through the process.

### 2.2 Data collection

The research data were collected through individual online interviews with physical education teachers in the study group. The interviews were conducted in Turkish using the Zoom app between December 10, 2024, and December 28, 2024, without any time breaks. No time limit was imposed to allow participants to express their experiences flexibly and in depth (the interviews lasted approximately 30–50 min), and the principle of data saturation—a fundamental criterion in qualitative research—was observed; as the data collection process progressed, it was observed that new ideas became very scarce and repetitions increased, reaching the point of data saturation. In accordance with research ethics principles, an Informed Consent Form was presented to all participants prior to the interviews. After the principles of data confidentiality and voluntariness were explained, both verbal and written consent was obtained from the participants before any audio recording was made. To ensure participants’ impartiality during the interviews, the interviews were conducted by a researcher who holds a bachelor’s degree in physical education, is currently pursuing graduate studies in the relevant field, but is not currently practicing as a teacher. This arrangement allowed participants to share their experiences more comfortably, without feeling peer bias or hierarchical pressure. The audio recordings of the interviews—conducted using a semi-structured interview form developed by the researchers—were transcribed verbatim for analysis. The development process of the semi-structured interview form followed these steps: (1) a review of the relevant literature; (2) drafting a pool of questions aligned with the research objectives; (3) revising the form based on feedback obtained from expert reviews by two academics specializing in qualitative research and physical education; and (4) finalizing the form by confirming the clarity of the questions through a pilot interview with two physical education teachers independent of the main study group.

### 2.3 Data analyses

The data analyzed in this study were obtained by re-examining a dataset from a related graduate thesis project, narrowing and refining its thematic structure to focus specifically on early-career challenges and adaptation. Following verbatim transcription of the interview audio recordings, an inductive content analysis method was applied. While the overarching domains of regional and psychosocial dynamics, PETE experiences, and stakeholder relationships broadly informed the initial structural focus, the specific codes, categories, and themes emerged entirely inductively through iterative open coding. Rather than imposing a rigid deductive framework, these three domains served as a natural thematic umbrella under which the inductively derived categories clustered.

Initially, two researchers independently read all transcripts multiple times to gain an overall sense of the data. Following this, the first researcher conducted initial open coding of meaningful units related to early-career challenges, and the second researcher independently reviewed the codes. Through repeated discussions as part of an established audit trail, the researchers filtered out elements outside the early-career focus, refined categories, and collaboratively developed the final themes manually without using software packages. In the final stage, they evaluated theme consistency and reached full consensus. To ensure anonymity, participants were assigned pseudonyms (e.g., PET1, PET2).

To illustrate the inductive mapping process, the following table presents a representative example (Table 2).

**Table 2:** An example of the inductive data analysis process.

Raw data	Representational code groups	Category	Theme
*“Transportation to the village school becomes a nightmare during winter months. Snow blocks the roads, and public transit runs only once a day. If I forget a simple piece of equipment in the city center, it takes weeks to get it delivered.”*	Geographical Isolation and Transportation Logistics	Physical and environmental constraints of the region	Regional and Psychosocial Dynamics

### 2.4 Trustworthiness

To ensure the qualitative rigor and trustworthiness of the study, we adopted Lincoln and Guba’s (1985) criteria of credibility, transferability, dependability, and confirmability. To enhance credibility and address researcher reflexivity, the research team maintained an ongoing awareness of their positionality. The first researcher’s background in physical education and sports—without a formal teaching appointment or active classroom experience—provided a theoretically informed yet objective perspective, while the second researcher oversaw the analytical framework in a supervisory and guiding role as a physical education teacher educator (PETE). To minimize potential interpretive bias, continuous reflexive discussions were maintained throughout the process. Furthermore, credibility and dependability were supported through researcher triangulation and consensus meetings, where the guiding researcher reviewed and steered the categories and thematic structures developed by the first researcher, ensuring that differing participant accounts were collaboratively evaluated against the raw transcripts. Participant quotations are also extensively used to ground the findings directly in the data. Transferability was strengthened by providing thick descriptions of the study context, participant characteristics, data collection, and analytical procedures, enabling readers to judge the applicability of the findings to similar settings. Confirmability was ensured by maintaining a secure audit trail of raw audio recordings, transcripts, and coding frameworks—retained confidentially in accordance with participant privacy—to demonstrate that the results faithfully reflect the collected data.

## 3 Findings

The findings of the study are organized under three principal themes: “regional and psychosocial dynamics,” “PETE and perceived professional competence,” and “stakeholder relations and perceived social support” (Table 3).

**Table 3:** Themes and codes (frequencies in parentheses).

Theme	Code
Regional and Psychosocial Dynamics	Climatic Conditions (12) Transportation (6) Importance Given to Education (5) Gender Disparity (5) Language Barriers (4) Clothing (3) Moral Norms (2)
PETE and Perceived Professional Competence	Teaching Practicum (Internship Process) (25) Adequacy of Courses in PETE (25) Practical Courses (21) Lack of Experience (18) Classroom Management and Organization (7) Assessment of Learning (5) Paperwork and Administrative Tasks (5) Lesson Planning (1)
Stakeholder Relations and Perceived Social Support	Giving Greater Importance to Academic Courses (21) A parent’s negative view of physical education class (19) Problems Experienced with School Administration (16) View of the Course as Free Time (15) Students’ Perception of the Course (15) Lack of Interest (7) Disallowing Extracurricular Activities (7)

### 3.1 Regional and psychosocial dynamics

The first theme, ‘Regional and Psychosocial Dynamics,’ reflects how climate, transportation, and local cultural characteristics shaped physical education teachers’ adaptation. The fact that newly appointed physical education teachers are predominantly assigned to Türkiye’s eastern and rural areas—which inherently present much harsher environmental and infrastructural conditions compared to central locations—leads them to face challenges that they have not previously experienced and undergo a difficult process of adaptation. The region-specific challenges encountered during and after this adjustment period can negatively affect teachers’ overall effectiveness. The combination of adverse climatic conditions with the lack of gymnasiums causes some physical education teachers in their early years of their career to conduct a large portion of their lessons in the classroom. While such physical and environmental difficulties may arise across various career stages, navigating them alongside the initial adjustment demands of professional entry appears to render this early phase particularly challenging for novice educators. The participants’ perspectives on the challenges arising from this process are as follows:

*“Winter is very long here. It is still cold in March, and we cannot take the children outside. There is a gym here, but it’s not heated, so we cannot go there in very cold weather.”* (PET2).

*“I work in a very hot region, and I have to take the children out to the yard to hold class. In our subject, students cannot benefit much or make progress in the classroom, so naturally, I have to go outside, and since I do not have any indoor space, the weather conditions challenge us a lot.”* (PET12).

*“I think the teaching part is only 30%, 40% at best— the rest is communication, you are trying to pull a rabbit out of a hat every day with a different scenario. In the freezing cold, we do not have a gym, no material support, we are trying to make do with what we have.”* (PET5).

*“The physical conditions are challenging for me. It is already a cold region climate-wise, and when schools open, the cold starts right from September, October, and November. I have only just started holding classes again for the last month, you know, since April—sometimes March is nice enough to go fully outside, but otherwise, over the 8–9 month school year, you are stuck inside for five months out of necessity. We do a lot of classroom activities, at least the children have a good time, but as I said, because there’s no gym, the children are necessarily deprived of the subject.”* (PET14).

Such accounts illustrate how adverse weather and limited transportation can pose significant challenges to lesson continuity and teachers’ psychosocial adjustment. In this context, some participants reported that instructional activities were disrupted because high temperatures began early and persisted for extended periods in the regions where they worked, whereas others indicated that, because they were located in areas with harsh winter conditions, they experienced substantial difficulties in conducting lessons during cold spells. Another region-specific challenge concerns physical education teachers, who, in the early years of their careers, are typically appointed to rural schools and therefore encounter problems in accessing the school or traveling to central locations where tournaments are held. The participants articulated their experiences regarding this situation as follows:

*“There are times when we are even at risk of not being able to get to school; we have no public transportation to the school. Although it is a village in the central district, there is no public transport. I think there was a service in previous years, but there was the following problem with it: the bus schedule did not align with the school hours.”* (PET16).

*“Competitions with my school are held in the city center, which is 60 km away. When I spoke with the principal, he said that there would be difficulties in arranging transportation services for going to and returning from the competitions in the future.”* (PET7).

*“My school is very far from the district center, we are 57 km away, so we cannot participate in things held in the district, whether competitions or other activities. This is not possible. There is no transportation, and I am unable to provide a vehicle.”* (PET12).

In addition, it was stated that for physical education teachers in the early years of their careers, challenges such as parental attitudes, language-related difficulties, and divergences in gender roles and moral norms also constitute challenges experienced within the framework of regional and psychosocial dynamics:

*“At the beginning of the term, we had difficulties regarding sportswear. Probably due to their culture or perhaps family pressure, wearing sports clothes is regarded as shameful around here.” (PET2).*

*“They were saying that they did not send their daughters to school. This was one of the first problems I encountered. For example, if I want to form a girls’ volleyball team, there are families who do not give permission…” (PET7).*

*“Girls and boys do not want to come together in the same environment, even if it is just for a game. In any case, the class sizes were very small, especially in the first months, whereas in physical education, we prefer to have a larger number of students for socialization. Children already have an established order, something that has been taught to them from a very young age, and this naturally originates from the parents and families in which they were born and raised. We are unable to overcome this.” (PET12).*

*“I have had problems with some of the villagers. They are used to seeing physical education teachers as male, so when they see a female physical education teacher, they approach us differently, whether it is about our clothing or us personally. Our mere presence can make them uncomfortable; the very fact that our gender is female leads them to adopt a harsher attitude towards us.” (PET16).*

*“In the Eastern Anatolia region, the rules of everyday life are somewhat stricter. Families are not very receptive to sports activities, and we have to persuade them to participate. We have experienced this particularly with our girls’ volleyball team. The students are very willing, determined, and they enjoy volleyball, but we have to persuade not only the students, but also their families.”* (PET19).

The participants’ accounts reveal that, rather than framing these local dynamics solely as cultural barriers, the challenges encountered in the early stages of a career in rural settings are deeply intertwined with traditional gender roles and social expectations. The tension experienced by teachers typically stems from the conflict between urban-centered professional socialization and the entrenched social norms of rural communities regarding modesty, movement in public spaces, and women’s participation in sports. To navigate this environment, teachers must look beyond the cultural differences apparent at first glance and recognize that the community’s hesitations often stem from protective family structures rather than active opposition; this underscores the need for context-sensitive relational strategies during the transitional phase of the early career. The language barrier—another aspect of regional and social dynamics—can, in some cases, present teachers with unique adaptation challenges. Particularly in the Eastern and Southeastern Anatolia regions, factors such as local language dynamics (the use of both Turkish and Kurdish) and the fact that students who have migrated may not have sufficient knowledge of Turkish can occasionally lead to temporary language confusion in some classrooms. Such local and exceptional circumstances can cause teachers to encounter certain obstacles when communicating with students, particularly during their initial period at their assignment location. Indeed, as one teacher noted, “*The school’s own challenges are actually related to its social environment; the place where I work is a bit further east. I’m assigned to Siverek, Şanlıurfa, which has a significant minority population, and the children here are experiencing some confusion regarding language. When I first arrived, there were students I could not understand, and I had trouble with this.”* (PET 5). These statements illustrate how language barriers with such regional and limited effects impact teachers’ experiences during their initial period.

Based on the findings, it is clear that the process by which physical education teachers early in their careers adapt to the physical and social conditions of the regions where they work involves significant challenges in terms of climate, transportation, and local cultural contexts. While geographic factors such as transportation and climate are a general source of difficulty for many newly assigned teachers, the fact that physical education classes are primarily held in schoolyards leads to more intense seasonal constraints in this subject area. Furthermore, the intersection of local cultural structures and social value systems with school expectations emerges as a distinct point of contextual friction for teachers. The community’s gender roles and traditional expectations regarding sports—particularly concerning girls’ participation—reflect deeply rooted social norms that require early-career to navigate cultural landscapes they are unaccustomed to, rather than signaling an absolute opposition. Although idealistic teachers initially experience a disconnect between their urban-focused training and these regional realities, their accounts also reveal active negotiation efforts—such as engaging in dialogue with families and the community—to foster broader acceptance of physical activities. This process of adaptation underscores that the adjustment experienced in the early stages of a career across different regional contexts is shaped not only by structural barriers but also by teachers’ ongoing efforts to bridge the gap between local cultural expectations and educational goals. The participants’ accounts suggest that this incongruity between regional realities and professional expectations creates a profound sense of professional challenge and operational constraint for early career teachers.

### 3.2 PETE and perceived professional competence

Another salient theme identified in this study pertains to the challenges arising from teacher competence and PETE quality. In evaluating the difficulties encountered during the early years of their professional practice, participants consistently emphasized their level of professional competence, particularly their lack of practical experience. Teachers who reported experiencing a pronounced period of uncertainty and disorientation upon entering the profession indicated that they experienced difficulties in areas such as lesson planning, assessment and evaluation, classroom management, and the delivery of specific instructional content. Selected excerpts from teachers’ accounts regarding the challenges experienced throughout the process are presented below.

*“One of the greatest difficulties I faced at the beginning was, first and foremost, my lack of planning, because to be honest, I always struggled when there was no ready-made plan. I go to school prepared, and suddenly it starts raining, and I immediately have to move the class indoors. The plan must be changed immediately and a new one implemented. In this regard, I strongly felt that I was inexperienced. Practice is very important; even if there is no problem with the theoretical part, everything ultimately depends on practice.” (PET1).*

*“I also realized that I did not have sufficient command of the scales used to assess subject matter learning. Not knowing how to construct an appropriate scale when preparing students’ exams was exhausting for me, but over time I began to learn by actively engaging in the process.” (PET4).*

*“I am an individual athlete, and this has caused me to fall short at certain points. For example, I do not have full command of team sports, such as football or volleyball. Of course, we know the rules, but I am frankly not very good at conducting these games. I can teach them, but I think I am lacking.” (PET6).*

*“As a teacher, I can say that I experienced difficulties in managing the class, particularly in crowded classrooms. While organizing them, the fact that some students are not interested in that particular game or sport branch—for instance, male students’ lack of interest when we are doing volleyball, or female students’ lack of interest when we are doing football—makes it challenging for me to engage them in the lesson. In this regard, I can say that I struggle with classroom management, especially because the classes are somewhat crowded.” (PET8).*

As seen in these accounts, the initial challenges faced by teachers are not uniform; rather, these challenges stem from a combination of practical and operational shocks experienced in crowded settings, gaps in subject knowledge, and classroom management obstacles. While teachers such as PET1 and PET8 immediately feel lost when faced with sudden environmental changes (such as changes in weather conditions) or students’ diverse interests, others, such as PET4, have noted that initial shortcomings in assessment and planning were gradually overcome through active participation and on-the-job experience. This situation demonstrates that the early stages of a teaching career are characterized by a dynamic learning curve; during this process, theoretical preparation combines with the practical realities of the school environment, enabling teachers to gradually overcome and make sense of operational challenges over time. The fact that some of these challenges relate to theoretical knowledge also underscores the importance of the undergraduate education component. Another salient finding of this study concerns the challenges experienced by early career teachers in relation to their workload. It was observed that physical education teachers, in particular, encounter difficulties in implementing core practices specific to their subject area because of the intensity of administrative paperwork. Teachers’ views on this issue are presented below:

*“Especially in my first month, I struggled with this: I found out there was a great deal of paperwork. I mean, we are supposed to focus entirely on teaching, but while dealing with so much paperwork, and as a novice who has just been appointed, there is also the candidacy process and so on, and with all this paperwork we no longer have time to devote to students or to plan lessons for them.” (PET10)*.

*“In the beginning, there were times when I fell behind the school program because I was dealing with the paperwork.” (PET1)*.

*“Every month, we are asked to submit different kinds of documents. While trying to complete these documents, we end up falling behind in our lessons.” (PET21)*.

The origins of the early career challenges encountered by physical education teachers are closely associated with the undergraduate education process. Teachers’ first professional experiences begin during their undergraduate studies. In this regard, the quality of undergraduate education and the experiences gained during this period appear to play a significant role in shaping the early years of professional practice and play a critical role in addressing the difficulties encountered. The views of teachers regarding the adequacy of their undergraduate education and their teaching practicum (internship) experiences are as follows:

*“If we look at the practical component of undergraduate education, I was not able to do an internship for a long period of time. My internship coincided with the pandemic period, and I could attend only for 2 or 3 weeks. Therefore, unfortunately, I was not able to gain much experience.”* (PET4).

*“In practice, I do not think we received sufficient training at university on issues such as class size, communication with students, and approaches based on gender differences. I believe we developed these more through experience, over time, and after entering the profession.” (PET15).*

*“I can say that the knowledge and experience gained during undergraduate education are highly insufficient, because physical education is a subject that should be largely practice-based, a field that needs to be learned through lived experience. Without practice, theoretical knowledge remains very abstract.” (PET24).*

*“In theory, we are well equipped as teachers, yes, but under normal circumstances we are supposed to learn the practical aspects during our internship. I am one of the teachers whose internship coincided with the pandemic; that is, if I remember correctly, I was only able to attend the internship for three weeks, and I could not benefit from it effectively anyway. Since the school where I did my internship was a centrally located school, its facilities were very good, so the answer to the question of how I could work in schools without such facilities remained at a theoretical level. When it came to practice, I was taken aback, and there were many times when I felt inadequate.” (PET25).*

*“Because there is a lack of equipment at the school, I experience difficulties in terms of how to transfer the education I received at university to the school. I know how to convey it to the students, but since there is no equipment, I have trouble figuring out how I can convey it to the students using different materials.” (PET9).*

It has been observed that teachers at the beginning of their careers undergo a distinctly challenging period of adjustment. Participants’ narratives suggest that, despite favorable perceptions regarding the effectiveness of theoretical coursework in undergraduate programs, the structural gap experienced in translating theory into practice may, when confronted with large class sizes and on-the-ground realities, undermine teachers’ perceived professional competence and create conditions conducive to difficulties in professional adaptation. However, as seen in the teachers’ accounts, this initial professional shock is rarely a permanent state of inadequacy; rather, it marks the beginning of a gradual, on-the-job learning process in which practical strategies and self-confidence are slowly developed through active participation in the school environment. In this context, while these coping mechanisms help teachers navigate day-to-day challenges, the underlying roots of these initial struggles continue to trace back to structural shortcomings in undergraduate preparation and practical field experiences.

### 3.3 Stakeholder relations and perceived social support

Another salient finding of the study is that some of the challenges encountered by physical education teachers in the early stages of their careers originate from internal and external stakeholders. Although many of these problems are not specific to the early years of the profession and reflect the broader challenges of the teaching profession, they are nonetheless important as they illuminate how teachers experience relational dynamics and perceive support during this initial and delicate phase of their careers. While experienced educators can draw on their established professional standing to overcome such interpersonal and administrative challenges, novice teachers typically experience these challenges more intensely due to their transitional status within the school. When these difficulties are examined in relation to internal stakeholders such as school administrators and students, the following statements by the participants are particularly noteworthy:

*“When I first started, the administration did not provide much support regarding equipment; they did not allocate much budget for our physical education classes. We tried to obtain the equipment through sponsorships and by pooling resources with friends.” (PET2).*

*“In our country, everyone thinks they are a physical education teacher. From the administration to other teachers, everyone tries to interfere with your lesson. I experienced this frequently, especially when I was a candidate teacher. For example, my school principal would come into my class and ‘give me advice’ in front of my students… The administration can suddenly assign you other responsibilities during your lesson hours. For instance, when I am on duty as a supervising teacher and the school buses arrive, they may ask, ‘Teacher, while you are teaching your lesson, could you also put the students who finish class early onto the buses?’” (PET6).*

*“At some schools, the student profile is truly very good; they are genuinely eager to learn. You want to do something, but then you come into conflict with the administration, which does not want to do it. For example, when we say to the administration, ‘Teacher, please do not cancel today’ or ‘We are going to a match, we need these resources, please arrange a bus for us,’ we encounter a lot of problems with such requests nowadays.” (PET13).*

Furthermore, participants’ accounts of administrative and institutional dynamics highlight the significant challenges faced in the early stages of their careers. As seen in the quotes, novice teachers may have to cope with administrative constraints such as a limited budget for supplies. Although this issue of insufficient materials may seem like a general problem, the novice teachers’ statements suggest that it is not a top priority. Furthermore, when external stakeholders or school administrators interfere with teaching processes or suddenly assign non-instructional responsibilities during class time (such as managing school transportation while on duty, as highlighted by PET6), the vulnerability of early-career teachers can become even more pronounced. Even if students are eager to participate, institutional barriers or administrative reluctance regarding requests for extracurricular activities can lead to friction (as stated by PET13). Rather than indicating persistent institutional neglect, these experiences demonstrate that early-career teachers must actively negotiate their professional boundaries and institutional roles as they establish their authority within the school hierarchy. Another salient finding of the study is that diversity in students’ readiness levels and their varied socio-cultural backgrounds emerge as a significant adaptation and alignment area for teachers during the early years of their profession. This dynamic appears to shape not only for the difficulties teachers encounter in classroom management and communication processes but also for their perceived professional satisfaction and emotional burden. Noteworthy accounts provided by teachers regarding these student-related experiences are as follows:

*“The students’ profile level was very low. This is not only in terms of lessons and their academic achievement but also in sports. Their initiative is very weak, and the students do not participate in the lessons at the level I would like.” (PET12).*

*“The children also have established habits, and we cannot easily change these habits. Initially, they did not want to learn anything different. The boys wanted to play football, the girls wanted to play volleyball, and apart from that, they had no other habits. They were not open to new activities or disciplines either. At the beginning of the term, we experienced difficulties regarding sports clothing.” (PET2).*

*“There is a perception that physical education lessons are free periods. The students often insist, asking, ‘Teacher, are you going to let us do whatever we want?’.” (PET6).*

*“Since very few physical education teachers are appointed, either paid teachers or, how should I say, teachers from other subject areas take the lessons, so the children end up being uninformed, in a sense…” (PET5).*

As seen in these quotes, challenges related to students may stem not from internal shortcomings but, on the contrary, from deeply ingrained habits, previous experiences limited to structured physical education, and the lasting effects of past inconsistencies in instruction (such as classes taught by teachers from other disciplines, as noted in PET5). Initial encounters with rigid role preferences (such as gender-segregated sports options, as emphasized by PET2) or the perception of physical education as an “elective course” (PET6) may be disappointing for novice teachers; however, these statements reflect an adjustment phase during which teachers must gradually guide students beyond the routines they are familiar with and reshape their expectations to understand the curriculum more comprehensively. As external stakeholders, parents’ attitudes toward the course and subject area may reflect the psychosocial challenges physical education teachers encounter during the initial stage of their professional careers. Shaped by the heterogeneity of the parent profile and their perceptions of the course, this dynamic emerges as a critical relational factor influencing teachers’ perceived external social support and their sense of professional status within the school. The key views expressed by participants regarding the difficulties they experienced in relation to parents are presented below:

*“There were times when parents told the school administration that their children had reduced their focus on other subjects and were concentrating more on our lessons, and they believed their academic achievement had declined. Over time, we changed this perception through our students’ successes in sports and academics, and we reached a point where parents became more open to being persuaded. In fact, later on the situation was almost the opposite: parents started coming to us to ask that their children be given opportunities to engage in sporting activities.” (PET3).*

*“There are requests from the administration and from parents either for lessons not to be conducted, or, if they are conducted, for them to be kept at a very low level of intensity. Parents make requests such as ‘my child should not run’ or ‘my child should not sweat.’” (PET6).*

*“Parents mostly want students to concentrate on the subjects that will be included in exams…” (PET8).*

*“With regard to physical education, of course, their interest is weak; parents place greater importance on theoretical subjects, and physical education is somewhat pushed into the background in this respect. I had a student, a girl, whom I wanted to prepare for a sports high school. We experienced some difficulties due to the parent; she told me she was under pressure not to go to sports, in other words, her family did not want her to attend. We had such problems with parents.” (PET20).*

As seen in these accounts, parents’ approach stems not only from a lack of understanding of the value of physical education, but is also deeply intertwined with broader systemic pressures—such as concerns about the exam system and overprotective parenting regarding physical exertion (such as requests to avoid fatigue or sweating, as noted by PET6 and PET8) that are part of broader systemic pressures. Furthermore, family reservations regarding a sports career (such as the family pressure experienced by PET20) generally reflect traditional protective structures rather than active opposition. Of course, it is also important not to overlook the fact that, at the heart of the matter, this subject is not given sufficient importance. However, as seen in PET3’s experience, these initial obstacles are not insurmountable; by maintaining consistent communication, demonstrating the holistic and academic benefits of sports, and highlighting students’ achievements, teachers can, over time, build a more supportive and collaborative relationship with parents. In conclusion, the attitudes and approaches of internal and external stakeholders—administrators, students, and parents—toward physical education classes and new teachers appear to constitute a key psychosocial dynamic in the challenges encountered during the early stages of a teacher’s career. The lack of administrative support frequently cited by participants, certain problematic student behaviors, and specific parent profiles that downplay the importance of the class can lead teachers to experience a sense of professional loneliness and lack of institutional backing. This situation, in turn, appears to contribute to a widening gap between the idealistic educational orientations of some early-career physical education teachers and the realities of the school context; this mismatch may become particularly pronounced during the early years of professional socialization, when institutional authority is still taking root.

## 4 Discussion

This study examined the early career challenges encountered by novice physical education teachers upon entering the profession and the reflections of these challenges on their professional lives using a qualitative approach. The findings indicate that teachers’ early career experiences are shaped around three main axes: (1) regional and psychosocial dynamics, (2) PETE and perceived professional competence, and (3) stakeholder relations and perceived social support. In this section, the findings obtained within the framework of these three main themes are discussed in relation to national and international studies in the literature and evaluated theoretically.

### 4.1 Regional and psychosocial dynamics

The findings of the study indicate that one of the most salient dimensions of the career-related challenges encountered by physical education teachers in the early years of their profession is the physical, geographical, and sociocultural conditions of the regions in which they are employed. The climatic conditions, transportation limitations, physical inadequacies, and local cultural structures of these regions tend to shape teachers’ professional practices and evolve into domains of constraint that complicate their psychosocial adaptation processes. Participants particularly emphasized that, in the absence of sports halls or indoor facilities, climatic conditions may severely constrain the conduct of lessons. Taşmektepligil et al. (2006) similarly demonstrated that adverse weather conditions, inadequate facilities/equipment, and overcrowded classes negatively affect the implementation of lessons. Consistent with the findings of the present study, recent literature also notes that extreme climatic conditions reduce instructional efficiency in schools and hinder learning processes; furthermore, this situation generates a need for flexible scheduling, alternative instructional approaches, and resilient infrastructure (Albina et al., 2025; Ameer and Ghannam, 2026; Factores, 2025; Newsome et al., 2023; Pitogo and Gallano, 2025; Venegas Marin et al., 2024). While such systemic and environmental constraints may create challenges at various stages of a career, encountering them at the very beginning of one’s career—that is, at a time when pedagogical routines and support systems are still taking shape—seems to make these challenges even more critical, particularly for beginning educators.

Another critical challenge faced by teachers who commence their careers in rural regions is the limited availability of transportation and the resultant logistical constraints. Tournament and sports event organization processes, which constitute a core component of the physical education discipline, transform into a serious issue of access and a source of administrative concern for teachers employed in schools located at a considerable distance from central settlements. A review of the literature indicates that Aydoğan (2011) identified inadequate transportation, insufficient facilities, and lack of materials as key physical conditions that disrupt instructional processes for physical education teachers in village schools. When the general challenges encountered in the early years of the profession are examined, it is noteworthy that this challenge is predominantly addressed in relation to the ways in which climatic conditions impede transportation (De Abreu et al., 2022; Gössling et al., 2023). However, the findings of the present study diverge from this general tendency in the literature in a distinctive manner. Specifically, the results demonstrate that professional difficulty is nourished not only by adverse weather conditions but also by the distance associated with the rural location of schools and the logistical responsibility of transferring students to sports and social activities, together with the psychosocial burden that this responsibility entails. Because initial appointments are predominantly made to rural and geographically isolated areas, these logistical and transport-related hurdles disproportionately characterize the early career phase. While experienced educators may possess the institutional familiarity to negotiate or mitigate such logistical hurdles, novice teachers often face them with limited institutional leverage, thereby amplifying their initial professional strain.

The rural and socioculturally distinct settings that constitute the first station of physical education teachers’ professional lives generate a marked “pedagogical mismatch” between teachers’ values and the cultural codes of the regions in which they work. Because it involves body language, freedom of clothing and movement, physical contact, and outdoor interaction, the field of physical education is among the disciplines that are most rapidly influenced by gender roles and cultural dynamics. Indeed, teachers encounter gender-based local resistance when trying to make sense of students’ in-class behavioral patterns, establish communication, and, in particular, ensure the participation of girls in lessons or extracurricular sports activities. Aydoğan (2011) concluded that female physical education teachers are more frequently exposed to stress-inducing problems related to gender. Göktaş et al. (2011) likewise emphasized that the sociocultural differences in the regions where teachers work affect the conduct of lessons and may negatively influence teacher performance. The restriction of girls, specifically in terms of gender, continues to be discussed in the literature in relation to cultural norms in the context of physical education classes and sports activities (Dennaoui et al., 2024; Rohozneanu, 2025; Tallat et al., 2025). Although not all studies report absolute parental opposition, it appears that particularly cultural norms, safety concerns, and academic priorities are cited as reasons that limit girls’ participation (Raveena et al., 2024; Ružbarská et al., 2021). Studies focusing on cultural differences in the specific context of physical education teachers confirm that these dynamics constitute a factor that complicate instructional processes (Flory and McCaughtry, 2011; Flory et al., 2023; Lee and Yi, 2025). Although navigating local norms and social expectations can be challenging for educators regardless of their level of experience, such cultural friction becomes particularly daunting during the transition to a new professional and social environment. Furthermore, given the nature of first-time assignments, novice teachers are more likely to be assigned to regions where different cultural norms are particularly pronounced.

### 4.2 PETE and perceived professional competence

Physical education teachers are expected to deliver highly efficient lessons with limited equipment within restricted timeframes and confined spaces. However, the findings of this study indicate that, within this complex context, participants’ perceived professional competence is challenged by the managerial and disciplinary crises they encounter, and they experience substantial difficulties in implementing effective instructional processes. Balaban et al. (2019) reported that the rate at which novice teachers confront classroom disciplinary problems reaches 75% and that beginning physical education teachers face a wide range of disciplinary violations in sports settings. A review of the broader literature likewise shows that classroom management and discipline problems experienced during the initial years of teaching constitute one of the principal domains of difficulty that constrain teachers’ perceptions of their competence (Admiraal et al., 2023; Shoval et al., 2010; Sözen, 2018).

Another critical finding emerging from the study concerns the pressure of the professional workload experienced by novice teachers as a result of intensive paperwork and bureaucratic procedures. Because physical education teachers receive no practical preparation during their undergraduate education for tasks such as document tracking, official correspondence, and administrative planning, they encounter a pronounced period of disorientation and time loss in the early career years. This situation prevents teachers from directing their limited time and energy towards high-quality lesson preparation, thereby adversely affecting the quality of pedagogical processes. Ensign et al. (2018) similarly state that a large proportion of beginning teachers struggle to balance their instructional responsibilities with administrative duties, and that this imbalance disrupts their planning processes. The literature also clearly shows that difficulties arising from paperwork, reporting, and non-instructional responsibilities are common experiences at the start of teachers’ careers (Admiraal et al., 2023; Chandran et al., 2022).

The origins of the early career challenges encountered by physical education teachers are closely linked to their undergraduate education and the resulting perceptions of competence. In this study, teachers who emphasized the decisive role of professional experience focused on two main shortcomings regarding the internship period: the limited opportunities for practicum due to pandemic restrictions and, more broadly, the qualitative and quantitative inadequacy of school experience/internship duration. In the context of the quality of undergraduate programs, while most participants acknowledged that theoretical courses were conducted efficiently, they highlighted a clear structural gap in the stage of translating theoretical knowledge into practice. Teachers stated that theoretical preparation alone was insufficient in the field and that this lack of practical experience led to a pronounced struggle, particularly in managing crowded classes and controlling outdoor spaces. A review of the literature shows that Uğraş et al. (2019) support the findings of the present study by reporting that, due to constraints such as limited time and low diversity of school placements, pre-service teachers’ practicum experiences are inadequate. Similarly, Zapatero Ayuso et al. (2021) drew attention to the disconnect between theory and practice in pre-service teachers’ undergraduate education. The findings clearly demonstrate how decisive theory–practice misalignment, the paucity of practicum in the curriculum, and the quality of internship experiences are for teachers’ perceived professional competence. Bezeau et al. (2020) confirmed that when physical education teachers are unable to establish coherence between theoretical knowledge and practical instruction, they cannot employ instructional strategies effectively. Recent studies have likewise indicated that this gap between theory and practice gives rise to serious psychosocial tension between teachers’ idealized professional self-perceptions and the realities of the school context (Stewart and Jansky, 2022).

This disjunction between theory and practice and the lack of applied experience in undergraduate education pave the way for teachers to experience a marked adaptation crisis when they first enter the profession. Indeed, teachers explicitly stated that they experienced a significant period of disorientation during the adaptation process in their early teaching years. This period of uncertainty experienced by teachers who have completed their undergraduate education and are stepping into the early phase of their careers is conceptualized in the literature as “reality shock.” The conflict between idealized professional expectations and the concrete realities of the field becomes even more challenging when the theoretical knowledge acquired at university does not find a direct counterpart in the school environment. In particular, in the early years of the profession, factors such as classroom management, discipline, in-class implementation of lessons, the institutional structure of the school, and role ambiguity are among the key elements that trigger this shock (Botha and Rens, 2018; Çakar and Boydak Özan, 2025; Dicke et al., 2015; Tran-Thanh, 2021). Consistent with this, participants in the present study reported that, although they considered themselves sufficiently equipped in terms of subject-matter knowledge within their discipline, they nevertheless encountered various difficulties in practical processes such as classroom management, organization, planning, and assessment and evaluation, and that they personally experienced this state of reality shock. However, as emphasized throughout the study, this initial professional shock and the corresponding theory-practice gap are rarely a static or permanent state of inadequacy; rather, they signify a learning opportunity inherent in a transitional period. As novice teachers actively confront the realities on the ground, they gradually develop practical coping mechanisms over time, refine their teaching strategies, and bridge the initial gap between idealized preparation and practical application; however, the fact that one aspect of this gap is linked to the quality of undergraduate education should not be overlooked.

### 4.3 Stakeholder relations and perceived social support

Another key finding emerging from the study is that the early career challenges faced by physical education teachers in their first years are not limited to physical and curricular factors but also stem from interactions with stakeholders inside and outside the school. The attitudes and expectations of this stakeholder network—comprising administrators, colleagues, students, and parents—towards the physical education course and the newly appointed teacher play a key role in shaping teachers’ perceived social support and adaptation processes. Indeed, Öter (2018) reported that teachers who seek to encourage students’ participation in school sports are unable to obtain the necessary social and logistical support from school administration in areas such as communication, transportation, health, budget, and safety during competition periods, and they also face negative reactions from both administrators and parents. This situation reveals the decisive role of school leadership in shaping both the effectiveness of physical education and sports processes and teachers’ psychosocial well-being. Underlying these negative administrative attitudes are chronic issues such as the low status of the subject within school culture, marginalization, professional isolation, and role conflict (Ensign et al., 2018; Gariglio, 2021; Westerlund and Eliasson, 2021). While experienced educators may have the professional reputation and institutional capital to protect themselves against such administrative shortcomings, new teachers often experience these shortcomings more acutely during their initial socialization process. Clearly, this does not constitute an absolute generalization, and it is also known that there are administrators who adopt a constructive and supportive stance in the process. However, because the present research specifically focuses on the difficulties encountered in the early period of teachers’ careers, the restrictive and obstructive effects of inadequate administrative support on the implementation of in-class and extracurricular activities have been highlighted.

At the level of internal stakeholders, another notable result of the study is that diversity in student profiles and negative student behaviors constitute key factors that complicate early career teachers’ psychosocial adjustment and teaching processes. Although students appear to be interested in physical education lessons, they tend—largely as a result of past learning habits—to perceive the class as a kind of “free time activity” and behave without regard for rules, which constitutes one of the main relational stressors in the process. The mismatch between the pedagogical approaches idealized by teachers in the first years of their careers and students’ expectations of freedom can lead to a pronounced conflict of roles and expectations between the parties involved. In today’s context, the changing student profile—with dynamics such as behavioral diversity, differing learning needs, and generational differences—represents one of the areas that most challenge the adaptation processes of newly appointed teachers (Halitoglu and Uzunboylu, 2025; Murphy, 2010). The literature frequently emphasizes that this shift in the student profile can influence processes such as classroom management, motivation, and teacher–student communication. Although, in line with the research focus, attention has been concentrated on negative student behaviors as constraining elements, the presence of students who display high motivation and actively participate in the course should not be overlooked.

Among out-of-school stakeholders, the tendency of some parents to regard the lesson merely as a “leisure-time activity” compels early career physical education teachers to engage in a constant effort to legitimize the pedagogical purpose of the course, while also leading them to experience a sense of professional challenge and lack of external support. This indicates that a significant portion of parents do not adequately embrace the lesson’s educational value for students’ holistic development. In this respect, Öter (2018) corroborated the findings of the present study by noting that parents keep physical education and sports lessons in the shadow of academic subjects and provide only limited support to the process. While participants’ accounts show that parents with a background in sports adopt a more constructive attitude towards tournaments and the course, they also reveal that the majority of parents maintain an approach centered on academic achievement. Similarly, Kocayiğit and Ekinci (2020) reported that parents perceive extracurricular sports activities as an additional financial burden, which negatively affects students’ participation. Consequently, relationships with parents do not merely constitute a communication barrier for teachers in the early stages of their careers; rather, changing parent profiles and expectations emerge as structural challenges that directly undermine teachers’ professional identity construction and levels of job satisfaction (Lundqvist et al., 2024). Taken together, these dynamics clearly demonstrate the decisive role of stakeholder interactions in the first years of teaching in shaping physical education teachers’ professional socialization and institutional adaptation processes. Moreover, because these interpersonal conflicts coincide with a period in which professional boundaries and institutional authority are still being negotiated, they appear to leave the early stages of a career uniquely vulnerable to interpersonal and structural pressures. However, as demonstrated by teachers’ efforts to highlight the value of physical education and encourage social participation over time, these relational barriers—as the statements suggest—are not insurmountable. Through active communication and goal-oriented collaboration, early-career teachers can gradually reshape stakeholders’ expectations and build more supportive professional networks.

## 5 Limitations and future research

Several limitations of this study should be noted. While structural challenges such as adverse climatic conditions, transportation barriers, and facility inadequacies can affect physical education teachers at various career stages, they are disproportionately experienced during the early career phase because initial appointments frequently place novice teachers in rural regions characterized by harsher climatic and environmental conditions. Consequently, rather than implying that these systemic difficulties are inherently exclusive to novice teachers, this study investigated how the intersection of these demanding conditions with early-career factors—such as pedagogical inexperience, low institutional status, and a lack of established support networks—compounds their impact, making them particularly consequential and disruptive during professional entry. Future research could explore how discipline-specific stressors and adaptation mechanisms vary across different career stages through broader psychological perspectives.

Moreover, the participant group consisted of 25 volunteer physical education teachers, many of whom worked in Eastern and Southeastern Anatolia. Therefore, the findings may not capture the full diversity of early career experiences in other regions or school types. Consequently, the early career challenges and adaptation processes experienced by teachers working in regions with different geographical conditions or sociocultural dynamics may vary. To address these contextual variations, prospective studies could incorporate cultural adaptation perspectives to comparatively examine how novice teachers navigate regional norms and psychosocial stressors in different geographical settings.

From a methodological standpoint, the adoption of a qualitative research design and the use of semi-structured interview techniques restrict the extent to which the findings can be generalized to the wider population. However, they contribute substantially to a nuanced and in-depth understanding of the phenomenon under examination. To complement these qualitative insights, future scholarship should adopt longitudinal designs that track physical education teachers continuously, starting from their undergraduate education and field practicum through their initial years of active service.

Finally, the findings also bear traces of certain period-specific disruptions in participants’ undergraduate education and internship processes (such as the COVID-19 pandemic and differences in curricula and institutional resources across universities). Therefore, teachers’ evaluations of their levels of professional readiness and their perceptions of self-efficacy are constrained by structural conditions specific to the period in question and/or the institution from which they graduated. Prospective tracking across this educational continuum would provide critical evidence on how structural disruptions during undergraduate training shape long-term professional readiness, self-efficacy, and occupational identity over time.

## 6 Conclusion

This study illuminates how early career physical education teachers navigate a complex and multilayered period of professional adjustment, shaped by the interplay of structural, pedagogical, geographical, and sociocultural dynamics. Rather than viewing early career challenges merely as a product of individual inexperience, the participants’ accounts highlight three interrelated contextual dimensions. A holistic analysis of the qualitative data illustrates how these domains of difficulty are experienced across the main themes of regional and psychosocial dynamics, PETE and perceived professional competence, and stakeholder relationships, alongside perceived social support.

First, owing to the outdoor-oriented nature of the subject, teachers described physical barriers such as unfavorable climatic and transportation conditions in assigned regions as factors placing them in a spatially vulnerable position. These physical constraints intersected with perceptual barriers, including local cultural detachment toward the subject, the reduction of physical education to mere “games and pastimes,” and its marginalization relative to academic courses. Additionally, traditional gender roles restricting female participation and entrenched local cultural codes shaped the interaction patterns among teachers, students, and parents, constructing a complex sociocultural sphere that teachers had to navigate to establish professional authority. From this perspective, prioritizing the construction of indoor sports facilities, particularly in geographically challenging regions, emerges as a key institutional response to alleviate the negative impacts of environmental constraints on instructional processes. Furthermore, to support the contextual adaptation of newly appointed teachers in distinct sociocultural settings—enabling them to negotiate these local barriers effectively over time—implementing contextualized mentoring mechanisms and addressing regional dynamics within pre-service teacher education appear to be highly beneficial.”

Second, participants felt a lack of pedagogical and operational skills (e.g., classroom management, organizational routines) in the face of these regional and cultural challenges; they closely linked this to the balance between theory and practice in their undergraduate education and the quality of their practicum experiences. Despite their strong theoretical preparation, participants stated that limited practical experience—due to constraints related to curriculum structure, teaching methods, the structure of practicum placements, or special circumstances such as the pandemic—formed the basis for their concerns about competence and the initial challenges they faced in the field. Furthermore, based on the views of some teachers, it was understood that these shortcomings gradually diminished over time through adaptive coping strategies developed with experience. Accordingly, to enhance pre-service teachers’ professional preparation, it is vital to diversify the PETE curriculum by integrating lessons on context-specific sociocultural challenges and providing sustainable experiences across diverse school profiles—such as overcrowded, disadvantaged, or resource-constrained settings—rather than relying on standardized practicum environments. Furthermore, to help teachers navigate bureaucratic procedures and teaching obstacles more easily, establishing formal mentoring partnerships with experienced local colleagues is strongly recommended.

Finally, it has been noted that these initial career challenges are exacerbated by the dynamics within the school ecosystem and a perceived lack of social support. Limited administrative support, students’ tendency to view physical education as unstructured “free time,” and parents’ performance-oriented attitudes have emerged as factors that pose challenges to teachers’ professional identity and the perceived value of this discipline during the early stages of their careers. However, the existence of examples showing that these relational challenges can be gradually overcome through active communication and the promotion of collaboration over time offers hope that this issue is surmountable. In this context, as a practical recommendation derived from the findings of this study and proposed by the author—although the direct benefits of this would require empirical evaluation in future research—starting the academic year with a collaboratively developed, partnership-based “School-Based Physical Activity Calendar” serves as a practical strategy to begin the academic year, reduce administrative distance, reshape parents’ preconceptions, and channel students’ energy toward structured educational outcomes; thereby fostering a participatory school culture.

In conclusion, the findings indicate that these three dimensions should not be considered in isolation from one another. Regional and cultural conditions create challenging environments, while the approaches of certain stakeholders can make these challenges even more taxing. The gap between theory and practice at the undergraduate level can sometimes put teachers at a disadvantage in coping with these challenges, turning the early stages of their careers into a critical transition and adaptation phase. However, as this study also emphasizes, these challenges in the early stages of a career are neither permanent nor insurmountable. Through active professional initiative, experience, and communication with stakeholders—however challenging that may be—new teachers strive to gradually bridge the gap between their initial preparation and the realities of the field. These findings emphasize that, rather than proposing a single prescriptive solution, mitigating these early-career challenges requires a two-pronged approach: at the macro level, enriching practical and field-based experiences within physical education teacher education (PETE) programs; and at the micro level, implementing functional, school-based mentoring and institutional support systems.

## Data Availability

The raw qualitative datasets generated and analyzed during the current study are not publicly available due to participant confidentiality and ethical restrictions. However, anonymized coding frameworks and further inquiries regarding the data are available from the corresponding author (Hidayet Suha Yüksel, hsyuksel@ankara.edu.tr) upon reasonable request.
